# Why We Need to Rethink the Diseases of Affluence

**DOI:** 10.1371/journal.pmed.0020104

**Published:** 2005-05-03

**Authors:** Thomas E Novotny

## Abstract

Novotny discusses the implications of a new study in *PLoS Medicine* examining the relationship between national income and risk factors for cardiovascular disease.

Most people believe that as societies advance economically they have higher levels of cardiovascular disease (CVD) and other noncommunicable disease (NCD) risks. However, a more detailed analysis of how parameters of economic development are associated with health outcomes as well as NCD risk factors is needed to inform local and global health policies. Such an analysis might dispel prejudices about the “diseases of affluence” and stimulate policy approaches and research that appropriately target emerging risk groups across the globe, regardless of socioeconomic status.

## From Intuition to Data

In a study in this month's *PLoS Medicine*, Ezzati and colleagues have taken a close look at population data available in 80 or so countries on body mass index (BMI), cholesterol, and hypertension [1]. They looked at how these CVD risk indicators are predicted by three broad economic parameters: national income, average share of household expenditure spent on food, and proportion of the population living in urban areas. This cross-sectional analysis drew on published studies, reports from ministries of health and the World Health Organization, data from household surveys, demographic data, and centrally available economic indicator data to model the relationship between the selected CVD risk factors and economic/demographic status.

The overall results suggest that average BMI and cholesterol increase with national income and then flatten out at higher incomes (or even decline), except in the United States, the home of the Big Mac and of leading practitioners of the sedentary urban lifestyle (in the US, BMI and cholesterol levels do not flatten out with higher income). Not surprisingly, there is an inverse relationship between BMI and proportion of household income needed for buying food in most countries.

Urbanization is associated with higher average income, and Ezzatti and colleagues found that urbanization is associated with higher BMI and cholesterol. As urbanization progresses and food availability equalizes among both urban and rural populations, there is less of an association between increasing income and increasing BMI and cholesterol. However, some persistently agricultural economies with large populations (such as Nigeria and Indonesia) tend to retain the inequities that influence diets, leading to protein-calorie deficiencies among those with lower income and increased BMI among those with higher income. Systolic blood pressure did not have as robust a relationship as BMI or cholesterol to urbanization or national income.

## Looking Ahead

Despite the limitations of multinational data and the broad brush approach used to interpret these data, there are some important lessons that emerge from this study regarding the population distribution of multiple CVD risk factors. These risk factors are systematically finding their way to low- and middle-income countries and the vulnerable populations therein that still suffer from childhood illness and high communicable disease burdens.

This shift is already having an effect on the epidemiology of CVD and other NCDs, particularly in middle-income countries. And we can predict that as low-income countries achieve economic growth, the disease burden of NCDs will be waiting for them as well. Effective policies can prevent some of this impact, if action is taken now; Ezzati and colleagues' work provides a basis for planning such interventions.[Fig pmed-0020104-g001]

**Figure pmed-0020104-g001:**
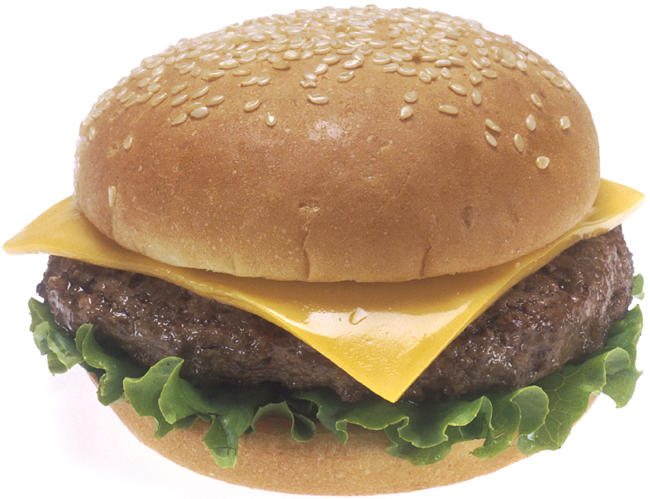
A shift towards “Western diets” high in saturated fats is occurring in developing countries (Photo: Renee Comet/National Cancer Institute)

## Planning for the Future

To target these interventions effectively, better data are needed on the prevalence of CVD risk factors in low- and middle-income populations, and on the association of these risk factors with NCD health outcomes. CVD risk factor surveillance should be incorporated into national program planning and into best practices for NCD control supported by the World Health Organization and other health development agencies. Multinational agreements, such as the recently activated Framework Convention on Tobacco Control (www.who.int/tobacco/en), can create effective international cooperative efforts to stem the tide of global tobacco use. Global tobacco control should be a health and foreign policy concern even for those countries that have not ratified the treaty.

As populations assume more of an urban lifestyle, they should not be limited in their choices for healthy foods, suffer from lack of safe water, or lose opportunities for physical activity. These problems can be reduced through good urban planning, better food policies, improved environmental engineering, and better attention to healthy lifestyle practices in our growing cities. Screening for hypertension, hypercholesterolemia, and nicotine addiction need to become a part of good clinical practices in low- and middle-income countries. Of course, screening for these risks should then also be accompanied by better availability of low-priced secondary prevention therapies such as generic versions of anti-hypertensives, statins, and nicotine replacement therapies.

## Getting the Balance Right

This is not to say that the big infectious disease killers and child health problems should be ignored. Rather, we need to learn from the history of socioeconomic development that it is not simply affluence that permits the increased impact of CVD and other NCDs; it is the risk factors for these diseases that spread across socioeconomic boundaries, causing the same illnesses regardless of the socioeconomic status of the population.

Increased attention should be paid to these diseases not just in the developed world, but also in the developing world, where the unfinished agendas on communicable disease and childhood illness have drawn the most attention. Addressing CVD risk factors could best be accomplished through improved international cooperation, better understanding of the risks of globalization, and development of appropriate research and technologies that apply to low- and middle-income populations.

## Abbreviations

BMI: body mass index
CVD: cardiovascular disease
NCD: noncommunicable disease
